# Rapid and simple detection of Tamiflu-resistant influenza virus: Development of oseltamivir derivative-based lateral flow biosensor for point-of-care (POC) diagnostics

**DOI:** 10.1038/s41598-018-31311-x

**Published:** 2018-08-29

**Authors:** Seul Gee Hwang, Kab Ha, Kyeonghye Guk, Do Kyung Lee, Gayoung Eom, Sinae Song, Taejoon Kang, Hwangseo Park, Juyeon Jung, Eun-Kyung Lim

**Affiliations:** 1 Hazards Monitoring BioNano Research Center, KRIBB, 125 Gwahak-ro, Yuseong-gu, Daejeon Korea; 2 BioNano Health Guard Research Center, KRIBB, 125 Gwahak-ro, Yuseong-gu, Daejeon Korea; 3 Department of Nanobiotechnology, KRIBB School of Biotechnology, UST, 217 Gajeong-ro, Yuseong-gu, Daejeon Korea; 4 Department of Chemistry, KAIST, 291 Daehak-ro, Yuseong-gu, Daejeon Korea; 5 Department of Bioscience and Biotechnology, Sejong University, 209 Neungdong-ro, Gwangjin-gu, Seoul Korea

## Abstract

We have developed a novel oseltamivir derivative (oseltamivir hexylthiol; OHT) that exhibits a higher binding affinity for Tamiflu-resistant virus (Tamiflu resistance) than for the wild-type virus (Tamiflu-susceptible virus; WT) as an antibody. First, OHT-modified gold nanoparticles (OHT-GNPs) are used in a simple colorimetric assay as nanoprobes for the Tamiflu-resistant virus. In the presence of Tamiflu-resistant virus, they show a colorimetric change from deep red to purple because of the OHT-GNP aggregation driven by strong interactions between OHT and neuraminidase (NA) on the surface of the Tamiflu-resistance. Moreover, the color gradually turns purple as the concentration of the Tamiflu-resistant virus increases, allowing the determination of the presence of the virus with the naked eye. Furthermore, an OHT-based lateral flow assay (LFA) has been developed as a rapid and easy detection device for Tamiflu resistance. It shows detection specificity for various virus concentrations of Tamiflu-resistant virus even for the mixture of WT and Tamiflu-resistant viruses, where the limit of detection (LOD) is 5 × 10^2^ ~ 10^3^ PFU per test (=1 × 10^4^ PFU/mL). It has been confirmed that this platform can provide accurate information on whether a virus exhibits Tamiflu resistance, thus supporting the selection of appropriate treatments using point-of-care (POC) diagnostics.

## Introduction

**Figure 1:**
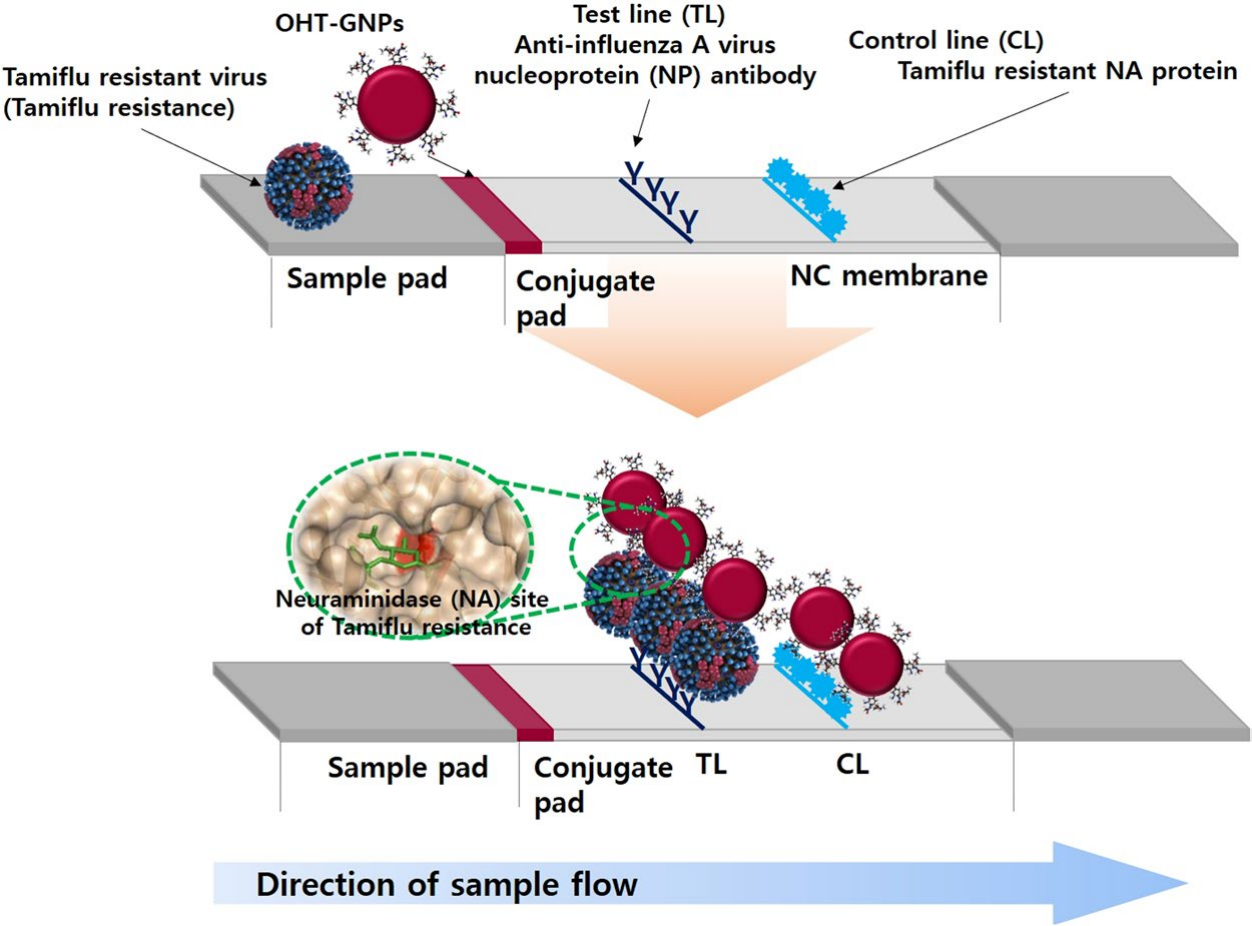
Schematic illustrations of the oseltamivir hexylthiol (OHT)-based lateral flow assay (LFA) for Tamiflu-resistant virus (Tamiflu resistance) detection as point-of-care (POC) diagnostics. OHT-GNPs are loaded onto the conjugate pad, and anti-influenza A virus nucleoprotein (NP) antibody and Tamiflu-resistant NA protein are lined onto this nitrocellulose (NC) membrane on the test (TL) and control line (CL), respectively. All pads are assembled to produce an OHT-based LFA, and then, virus-containing buffer (1% BSA and 0.2% Tween20 in PBS) is dropped onto the LFA sample pad at room temperature. Detailed experimental conditions are described in the Methods. After 10 min, when Tamiflu resistance is detected, the test and control lines (TL and CL) are observed.

**Figure 2:**
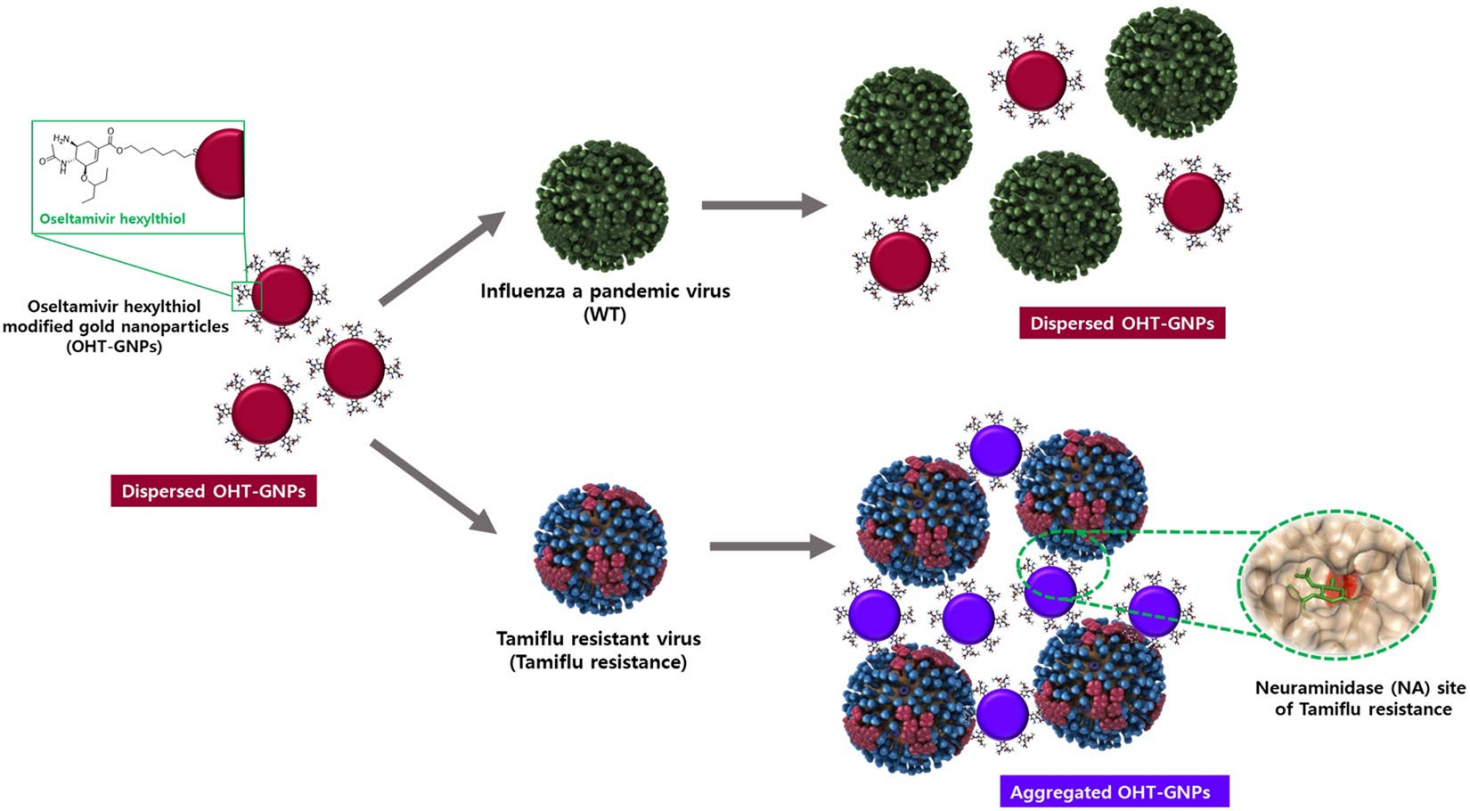
Schematic illustration of colorimetric assay for Tamiflu resistance detection using OHT-modified gold nanoparticles (OHT-GNPs). The color of OHT-GNPs changes from deep red to purple due to the binding of OHT to Tamiflu-resistant virus.

In 2009, the pandemic influenza A (H1N1) 2009 (pH1N1) virus emerged and circulated globally. In the early stages, most cases were responsive to antiviral neuraminidase (NA) inhibitors. NA inhibitors block the active site of the viral NA enzyme, preventing the cleavage of terminal sialic acid residues on the membrane of the infected cell and thus hampering viral propagation. Although the original circulating pH1N1 remained susceptible to NA inhibitor treatment, a drug-resistant pH1N1 appeared (<5%) because of increasing selective pressure resulting from the widespread use of NA inhibitors (particularly oseltamivir) for pandemic control^1–3^. According to the Centers for Disease Control and Prevention (CDC) and the World Health Organization (WHO), oseltamivir-resistant pH1N1 emerged and quickly spread around the world as a result of a histidine-to-tyrosine mutation at position 275 (N1 numbering; H274Y in N2 numbering) of viral NA^4–7^. In 2011, a notable increase in the occurrence of oseltamivir-resistant pH1N1 in patients who had not received oseltamivir treatment was noted; i.e., patients with oseltamivir-resistant virus (Tamiflu resistance) appeared irrespective of selective drug pressure^7–9^. Tamiflu-resistant virus-infected patients experienced adverse effects and even clinical failure despite the early initiation of treatment^10^. Controlling Tamiflu resistance has become a major public health issue, and appropriate treatment requires accurate virus information. In clinics, Tamiflu resistance is identified using genotypic and phenotypic assays^11^. Phenotypic assays include enzymatic assays and plaque reduction assays, which are used to assess resistance to NA inhibitors^12^. Because these assays require additional growth time to obtain sufficient quantities of isolated virus from patients, genotypic assays (e.g., polymerase chain reaction (PCR) and pyrosequencing, DNA sequencing and nucleic acid hybridization) are preferred to phenotypic assays in many laboratories and clinics, as these assays enable direct detection with high throughput and sensitivity^13–20^. However, these assays are ill-equipped for use as point-of-care (POC) diagnostics because they require trained personnel, time-consuming procedures and additional analytical equipment^21,22^, We suggest novel rapid diagnostic methods using an oseltamivir-based molecule to simply and rapidly detect Tamiflu resistance (Fig. 1)^23^. Herein, we first developed OHT-modified gold nanoparticles (OHT-GNPs) as a colorimetric probe to detect Tamiflu resistance with the naked eye using a color change from deep red to purple in the presence of the Tamiflu-resistant virus (Fig. 2)^24–30^. Furthermore, by using the OHT-GNPs, we fabricated OHT-based lateral flow assays (LFAs) as POC devices based on the specific interaction between OHT and the Tamiflu-resistant virus^31–46^.

## Materials and Methods

### Materials

Gold(III) chloride (HAuCl_4_) solution and sodium citrate were purchased from Sigma-Aldrich (USA). An NA-Fluor^TM^ Influenza NA assay kit and a Viral RNA extraction kit were purchased from Applied Biosystems (USA) and Qiagen (Germany), respectively. Absorbent pads (CFSP203000, cellulose fiber) and nitrocellulose (NC) membrane (HF090MC100) related to lateral flow assay strips were purchased from Millipore (USA). A conjugate pad (polyester pad) was purchased from Boreda Biotech (Korea). Anti-influenza A virus nucleoprotein (NP) antibody and influenza A H1N1 neuraminidase/NA (H274Y mutation) as a Tamiflu-resistant NA protein were purchased from Abcam and Sinco Biological Inc., respectively. All other chemicals were of analytical grade and used without further purification. Pandemic H1N1 virus (A/California/07/2009) (WT) was provided by the BioNano Health Guard Research Center (H-GUARD). Pandemic H1N1/H275Y NA-mutant virus (H275Y mutation A/Korea2785/2009 pdm: NCCP 42017) (Tamiflu resistance) isolated from Gyeongsangnamdo in Korea was obtained from the National Culture Collection for Pathogens (NCCP), which is operated by the Korea Centers for Disease Control and Prevention. All virus titers were determined by real-time PCR using a GoTaq^®^ 1-Step RT-qPCR System (Promega, USA) according to the manufacturer’s instructions.

### Synthesis and characterization of oseltamivir hexylthiol (OHT)

Supplementary Fig. S1(a) shows the OHT synthetic scheme. First, S-6-hydroxyhexyl ethanethioate (2) was synthesized. Potassium thioacetate (CH_3_COS^−^K^+^) (6.31 g, 55.2 mmol) was added slowly to a stirred solution of 1-bromohexanol (1) (5.00 g, 27.6 mmol) in anhydrous dimethylformamide (DMF, 50 mL) at room temperature. The solution was stirred for 12 hr and diluted with 30 mL of deionized water. Next, the reaction mixture was extracted with ethanol three times. The organic layer was dried over anhydrous MgSO_4_, filtered, and concentrated. This concentrate was purified using column chromatography, and then, pale-yellow liquid *S*-6-hydroxyhexyl ethanethioate (2) was obtained with a yield of 86% (4.20 g). Second, di-tert-butyl dicarbonate (Boc_2_O) (7.84 mL, 34.1 mmol) and triethylamine (Et_3_N) (6.80 mL, 483.8 mmol) were added to a stirred solution of oseltamivir phosphate (3) (10.0 g, 24.4 mmol) in MeOH (50 mL) at room temperature to synthesize (3R,5S)-ethyl 4-acetamido-5-(tert-butoxycarbonylamino)-3-(pentan-3-yloxy)cyclohex-1-enecarboxylate (4). This mixture was stirred for 12 hr at room temperature, and then, deionized water (H_2_O) (100 mL) was added with continued stirring. After 1 hr, the generated product (white powder) was obtained by filtering and washing with deionized water. The purified product was dried in a vacuum oven to obtain a white solid of carbamate (4) with a 56% (5.71 g) yield. Third, to synthesize (3R, 5S)-4-acetamido-5-(tert-butoxycarbonylamino)-3-(pentan-3-yloxy)cyclohex-1-enecarboxylic acid (5), NaOH (663 mg, 16.6 mmol) was added to a stirred solution of (4) (5.70 g, 13.8 mmol) in tetrahydrofuran (THF)/H_2_O (10:1, v/v) (30 mL). This mixture was stirred for 24 hr and then concentrated to remove the reaction solvent. The concentrate was diluted with 20 mL of deionized water and cooled to 0 °C. The pH of this mixture was adjusted to pH 5 using 1 M aqueous HCl, and the mixture was stirred for 1 hr. The generated white powder was filtered, washed with deionized water, and then dried in a vacuum oven. A white powder of (5) was obtained with a 75% (4.0 g) yield. Fourth, (5) (4.0 g, 10.4 mmol) was mixed with CH_2_Cl_2_ (30 mL), and (2) (2.2 g, 12.5 mmol), 1-ethyl-3-(3-dimethylaminopropyl) carbodiimide hydrochloride (EDC·HCl) (2.8 g, 14.6 mmol), 4-(dimethylamino)pyridine (DMAP) (1.5 g, 12.5 mmol), and triethylamine (Et_3_N) (2.9 mL, 20.8 mmol) were added into the (5) mixture under vigorous stirring at room temperature. After stirring for 24 hr, this mixture was added to deionized water and extracted three times with CH_2_Cl_2_ (30 mL). The organic layer was dried over anhydrous MgSO_4_, filtered and then concentrated. This concentrate was then purified using column chromatography (hexane-ethyl acetate), and a colorless liquid of (6) was obtained with an approximately 62% (3.5 g) yield. Finally, we synthesized oseltamivir hexylthiol (OHT) (7). HCl (2.15 mL, 25.8 mmol) was slowly added into a stirred solution containing 3.5 g of (3R,5S)-6-(acetylthio)hexyl-4-acetamido-5-(tert-butoxycarbonylamino)-3-(pentan-3-yloxy)cyclohex-1-enecarboxylate (6) (6.5 mmol) dissolved in MeOH (30 mL) at room temperature, and then, the mixture was heated to 50 °C. After 72 hr, this solution was cooled to room temperature and concentrated to remove the reactant solvent. This concentrate was diluted with MeOH (5 mL), and then, diethyl ether was slowly added under stirring. The generated white powder was filtered and washed with diethyl ether. The filtered product was dried in a vacuum oven to afford OHT (7) as a white powder with a 20% (560 mg) yield. The characteristic band of OHT was confirmed using proton nuclear magnetic resonance (^1^H-NMR) (600 MHz) (Inova 600NB, Varian) with CD_3_OD as the solvent. An asterisk (*) indicates the thiol group (-SH) in OHT (Supplementary Fig. S1(b)).

### Sequencing of the viral NA gene of viruses

**Figure 3:**
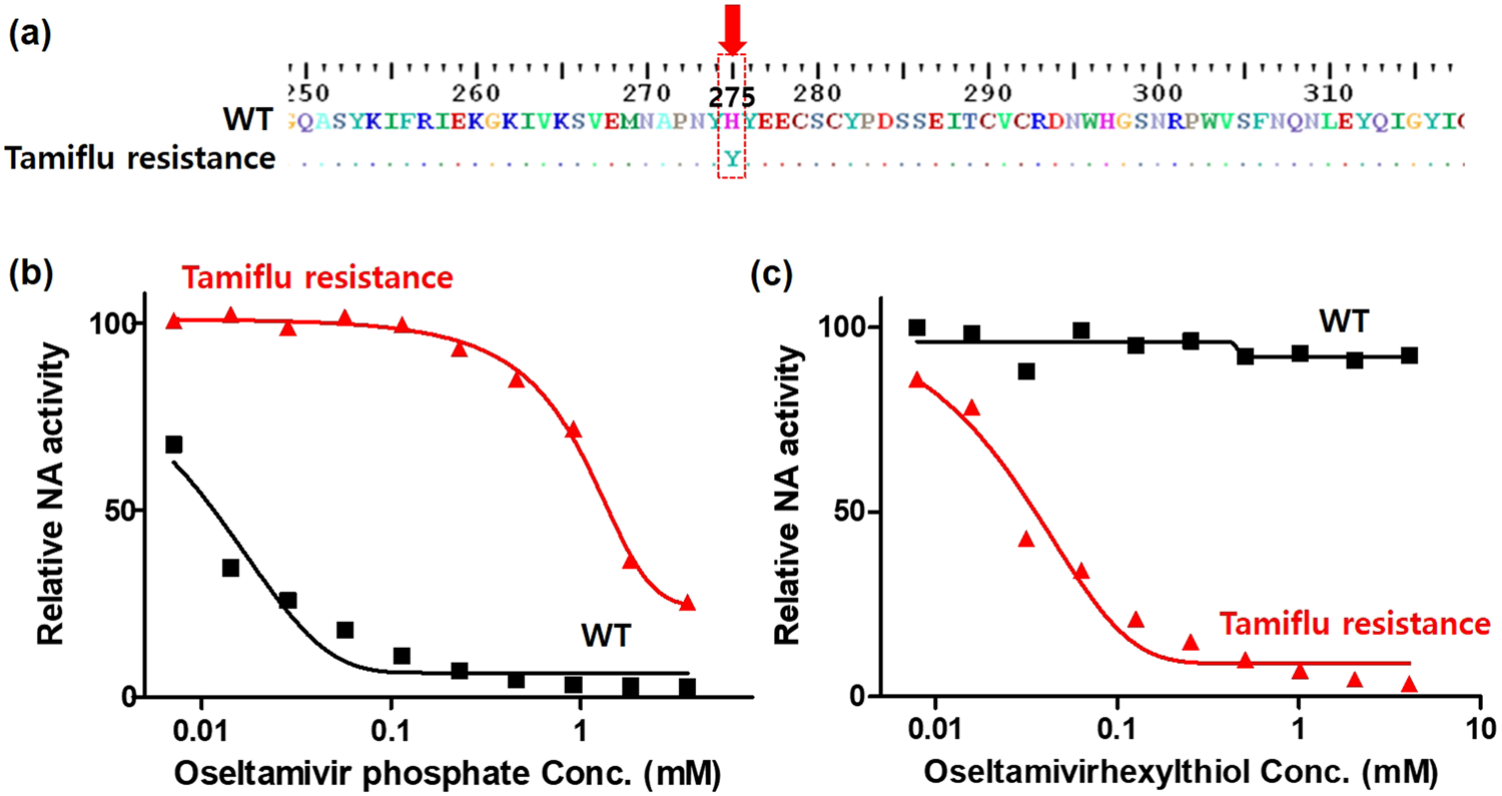
(**a**) Sequence alignment of NA genes from the WT and Tamiflu-resistant viruses to confirm the genotype at position 275, performed using one-step RT-PCR. The red arrow indicates the mutated amino acid in Tamiflu-resistant NA compared with WT NA when histidine (H) at position 275 is replaced by tyrosine (Y). NA inhibition tests of (**b**) oseltamivir phosphate (Tamiflu^TM^) and (**c**) oseltamivir hexylthiol (OHT) for the WT (black) and Tamiflu-resistant enzymes (red) using a commercial fluorescence-based NA inhibition assay kit. The relative NA activity was calculated from the fluorescence intensity ratio of treated to nontreated virus (0 mM) and the average values were plotted (n = 3).

The NA gene in virus preparations was sequenced to confirm the genotype at position 275. Viral RNA was extracted from the WT and Tamiflu-resistant viruses using a viral RNA extraction kit according to the manufacturer’s instructions. Purified RNA was reverse-transcribed using a one-step RT-PCR kit, and PCR was performed with specific primers. One-step RT-PCR for the NA gene was performed using BIOFACT^TM^ one-step PCR master mix. The RT-PCR conditions for the NA gene amplification were as follows: reverse transcription at 50 °C for 30 min; initial PCR activation at 95 °C for 15 min; 35 cycles of 95 °C for 20 sec (denaturation), 54 °C for 40 sec (annealing) and 72 °C for 1 min (extension); and a final extension at 72 °C for 5 min^47,48^. The amplified PCR product was then subjected to RT-PCR in the same reaction tube, and the conserved regions were identified by generating multiple sequence alignments using BioEdit software to confirm both the presence of the desired mutations and the absence of additional mutations. The viral phenotype was confirmed by pyrosequencing the NA gene (WT and Tamiflu-resistant) (Fig. 3 (a)).

### NA enzymatic inhibition assay for OHT

The NA enzymatic inhibition activity assay was conducted using an NA-Fluor^TM^ Influenza NA assay kit according to the recommended protocol to evaluate the NA inhibition effect. In this assay, 2-(4-methylumbelliferyl)-a-D-N-acetylneuraminic acid (MUNANA) was used as the fluorescent substrate to determine the NA enzymatic parameters. Briefly, 10^6^ PFU/mL of each virus (WT and Tamiflu-resistant) was plated in a 96-well plate, and then, various concentrations of OHT were added to each well. After incubation for 30 min at 37 °C, the NA-Fluor^TM^ substrate was added to each well. The reaction was terminated after 60 min at 37 °C by adding the stop solution, and the fluorescence was measured with excitation at 360 nm and emission at 450 nm. The relative NA enzyme inhibition was determined as the ratio of the fluorescent intensity of each virus incubated with OHT to that of the nontreated virus, which was considered to be 100% active. The tests with oseltamivir phosphate (Tamiflu^TM^) were performed in the same manner as the controls. Based on these NA inhibition values, the half-maximal inhibitory concentration (IC50) was calculated by the Prism software.

### Computational methods for OHT binding analysis

Three-dimensional (3D) atomic coordinates were prepared using the X-ray crystal structures of the WT and Tamiflu-resistant NA complexed with oseltamivir (PDB code: 4B7Q)^49^ as the receptor models for the docking simulations of OHT. The Gasteiger-Marsilli atomic charges^50^ were assigned for all of the protein and ligand atoms to calculate the electrostatic interactions in the protein-ligand complexes. Docking simulations were then conducted using the AutoDock software^51,52^ to calculate the binding modes of OHT to the WT and Tamiflu-resistant NA. Of the 20 OHT conformations generated in the docking simulations, those clustered together had binding modes differing by less than 1.5 Å in positional root-mean-square deviation. The most-stable binding configuration in the top-ranked cluster was selected for further analysis.

### Preparation of OHT-GNPs

First, 1 wt% sodium citrate was dissolved in 100 mL of deionized water under vigorous stirring at 95 °C, and then, 1 mL of 1 wt% gold(III) chloride (HAuCl_4_) solution was quickly injected into the solution mixture. This mixture was stirred at 95 °C until the solution color changed from yellow to deep red. The product solution was then cooled naturally to room temperature without being disturbed. The morphologies of the GNPs were determined using high-resolution (HR)-TEM (Tecnai^TM^ G2 F30) accelerated at 300 kV, and their crystallinities at 298 K were measured using a high-resolution powder X-ray diffractometer (XRD) (D/MAX-2500). After that, for OHT-GNP, 5 mL of GNP solution (1.4 mg/mL) was centrifuged at 17,000 rpm for 30 min to remove excess sodium citrate and then redispersed in 1 mL of deionized water. After repeating this procedure three times, the resulting gold solution and 10 mg of OHT dissolved in 1 mL of deionized water were vigorously mixed overnight at room temperature. This mixture was then centrifuged at 17,000 rpm for 30 min to remove unbound OHT molecules and resuspended in 1 mL of deionized water, affording OHT-GNPs with a deep red color. Then, the absorbance spectrum and surface analyses of GNPs and OHT-GNPs were carried out using a UV-Vis spectrophotometer (DU®800 Spectrometer) and an X-ray photoelectron spectrometer (XPS) (Theta Probe AR-XPS System, Thermo Fisher Scientific), respectively. The percent weight (%) of OHT in OHT-GNPs was analyzed using a thermogravimetric analyzer (TGA) (SDT-Q600, TA instrument).

### Colorimetric detection of Tamiflu resistance using OHT-GNPs

To confirm whether the OHT-GNPs could be used to selectively detect the Tamiflu-resistant virus over the WT in a colorimetric assay, the OHT-GNPs were first tested against the Tamiflu-resistant NA protein. First, Tamiflu-resistant NA protein was added to a 96-well plate and treated with the OHT-GNPs. After 5 min of treatment, a color change was observed and measured using a multidetection microplate reader (SpectraMax M2e, Molecular Devices). Second, Tamiflu-resistant virus was added to a 96-well plate and treated with OHT-GNPs. Its color change was observed and measured with the multidetection microplate reader. As a control, WT NA protein (Sino Biological Inc.) and WT virus were analyzed in the same manner.

### OHT-based lateral flow assay (LFA) for Tamiflu resistance detection

LFA strips consist of four components: the sample, absorbent pads, nitrocellulose (NC) membrane and conjugate pad. First, both the sample and absorbent pads were saturated with buffer containing 1% BSA and 0.2% Tween20. Anti-influenza A virus nucleoprotein (NP) antibody and Tamiflu-resistant NA protein were lined onto this membrane on the test (TL) and control lines (CL), respectively, and dried for 1 hr at room temperature. The conjugate pad was saturated with pretreatment buffer (1% PVP, 0.5% S10G) and then dried for 2 hr at room temperature. OHT-GNPs were resuspended in borate buffer containing 1% BSA and 1% sucrose and loaded onto the saturated conjugate pad. Finally, the pads were assembled in an overlapping sequence, using the adhesive tape at both ends of the NC membrane, and were cut to a width of 4 mm. Prepared LFA strips were stored with a desiccant at 4 °C until use. Tamiflu-resistant virus (50 μL) was mixed with 50 μL of dilution buffer (1% BSA and 0.2% Tween20 in PBS), and 100 μL of this solution was dispensed onto the LFA sample pad at room temperature. The appearance of the test and control lines (TL and CL) was monitored for 10 min. As a control, we used WT virus and only the dilution buffer (blank) without the Tamiflu-resistant virus. The OHT-based LFA was also evaluated in virus-containing 10% nasal fluid (Lee Biosolution, Inc.) as well as in virus mixtures prepared by mixing Tamiflu-resistant and WT viruses at various ratios in the dilution buffer (100 μL) in a same way as described above.

## Results and Discussion

### Characterization of OHT against Tamiflu-resistant NA

Oseltamivir (Tamiflu^TM^), approved by the Food and Drug Administration (FDA) in 1999, is a very effective drug for treating influenza patients at the early stage of infection. This drug is directly hydrolyzed to oseltamivir carboxylate, an active metabolite, and binds to NA-active sites, causing NA inhibition^11,53,54^. Oseltamivir prevents the release of progeny virus by cleaving the terminal sialic acid on glycosylated hemagglutinin (HA), blocking NA enzyme activity and thereby inhibiting the progression of the infection^55–59^. In addition to the strong hydrogen-bond interactions with the polar amino acid residues, the NA inhibitor also establishes hydrophobic contacts with the conserved residues in the viral NA active sites. Typically, Tamiflu-resistant pH1N1 viruses feature a histidine (His)-to-tyrosine (Tyr) substitution at position 275 of the NA protein, i.e., a His275Tyr (H275Y) NA mutation (N1 numbering)^60–62^. With the H275Y NA mutation, a conformational change occurs at the NA inhibitor binding site, resulting in inhibition of oseltamivir binding and conferring the highest level of resistance to oseltamivir. As a result, this mutant virus (Tamiflu-resistant) is approximately 1500-fold less sensitive to the drug than the pH1N1 virus (WT)^1–3^. Therefore, we predicted that an oseltamivir derivative could be useful to detect Tamiflu resistance and thus developed several oseltamivir derivatives by introducing organic molecules at each ester site in oseltamivir phosphate^1,2,11,12,63–67^. In particular, we determined that this group considerably affects binding to the NA protein since the ester in oseltamivir is hydrolyzed to a carboxylate when the drug is converted to the active metabolite. Thus, to develop a targeting moiety capable of nanoparticle surface modification, oseltamivir hexylthiol (OHT) was synthesized by linking a hexylthiol molecule to the ester groups in oseltamivir phosphate (Supplementary Fig. S1). First, we evaluated the ability of OHT to act as an NA inhibitor by binding to the NA site of the virus compared to oseltamivir phosphate by measuring the inhibition of NA enzymatic activity (Fig. 3(b,c))^68^. This assay indirectly measured the viral NA enzyme release via the fluorescence of methylumbelliferone, generated by the action of released NA on the substrate MUNANA^69^. As expected, an increase in oseltamivir phosphate concentration was accompanied by its binding to WT NA and inhibition of enzymatic activity. However, it did not show an inhibitory activity against Tamiflu-resistant NA (Fig. 3(b)). As shown in Fig. 3(c), OHT was a more potent NA inhibitor against Tamiflu-resistant NA, with an IC50 value of approximately 50 μM for NA inhibition, than against WT NA. These results were attributed to strong OHT binding to the NA site of the Tamiflu-resistant enzyme. To verify OHT binding to the NA site, we carried out additional docking simulations with extended three-dimensional (3D) grid maps to include the entire NA protein structure. The stable binding modes of OHT to both WT and Tamiflu-resistant NA proteins were obtained from docking simulations (Supplementary Fig. S2)^49–51^. OHT appeared to be stabilized at the active site of Tamiflu-resistant NA and exhibited a binding free energy (∆G_bind_) value lower than that for the WT. Consistent with the NA activity assay, OHT had a lower ∆G_bind_ value for the Tamiflu-resistant than for the WT enzyme by 3.3 kcal/mol, corresponding to a 250-fold higher binding affinity for the mutant NA. The terminal pentyl group of OHT exhibited different *van der Waals* interactions with the side chain of the NA residue 275 of the WT and H275Y mutant. The minimum distance of the OHT pentyl moiety to the side-chain phenyl ring of Tyr275 is less than 4.45 Å in the active site of Tamiflu-resistant NA, but it is 5.33 Å in WT NA. Because the OHT-NA interactions were similar for the WT and Tamiflu-resistant NA, except for the pentyl group of OHT, the hydrophobic interaction of Tyr275 with the pentyl part of OHT enhanced the binding capacity of OHT to the Tamiflu-resistant NA compared with WT NA. The binding mode shown in Supplementary Fig. S2 seems to be specific to OHT in the sense that it is difficult for oseltamivir to interact directly with His275 because the neighboring Glu276 moves the hydrophobic pocket away from the inhibitor^52^. The alkyl chain appears to bind tightly to the channel connecting the protein surface and the active site, which has the effect of keeping the terminal pentyl moiety in proximity to His275. The induction of the binding mode change by alkylation may thus be invoked to explain the selective binding of OHT to the H275 mutant NA site. Based on modeling and experimental studies, OHT was developed as an efficient and selective target moiety to detect Tamiflu resistance.

### Characterization of OHT-GNPs

We attempted to directly detect Tamiflu resistance with the naked eye. For OHT-GNP preparation, first, aqueous-phase gold nanoparticles (GNPs) were synthesized using the citrate-reduction method, in which a gold salt (HAuCl_4_) was reduced by sodium citrate as the reducing and capping agent^64^. GNPs were similar-sized, with diameters of 12 nm (12.7 ± 2.2 nm), were almost spherical shapes and had good monodispersity (Supplementary Fig. S3(a)). Their crystallinities were assigned based on diffractions from the (111), (200), (220), and (311) planes, which corresponded to the face-centered cubic (fcc) facets of gold (Supplementary Fig. S3(b))^70^. Subsequently, OHT-GNPs were generated by forming a strong covalent Au-S bond between OHT and GNPs (14.4 ± 1.5 nm). GNPs had a maximum absorbance at 520 nm and were deep red in color. OHT-GNPs still remained well-monodispersed in the aqueous phase and maintained the deep red color without aggregation (Supplementary Fig. S3(c)). Both surfaces predominantly exhibited carbon (C) and oxygen (O) element spectra. Whereas bare GNPs clearly showed a sodium (Na) spectrum corresponding to sodium citrate as a stabilizer of GNPs in the range of 400–410 eV (7.32%), OHT-GNPs hardly showed the Na peak (0.48%). Moreover, the atomic percent (At.) of nitrogen (N) in OHT-GNPs was increased (4.73%), corresponding to the OHT, compared with that in GNPs (1.15%). It was confirmed that OHT was well-modified on the surface of GNPs instead of sodium citrate (Supplementary Fig. S3(d,e))^71–73^. We also determined the approximately 46.1 wt% of the OHT layer and 53.9 wt% of the GNPs in OHT-GNPs (1.7 mg) from TGA (Supplementary Fig. S4); in other words, there were approximately 0.78 mg (0.7837 mg) of OHT and 0.92 mg of bare GNPs. Thus, it was estimated that approximately 1.94 μmol of OHT coated 1 mg of GNPs.

### Colorimetric detection of Tamiflu resistance using OHT-GNPs

**Figure 4:**
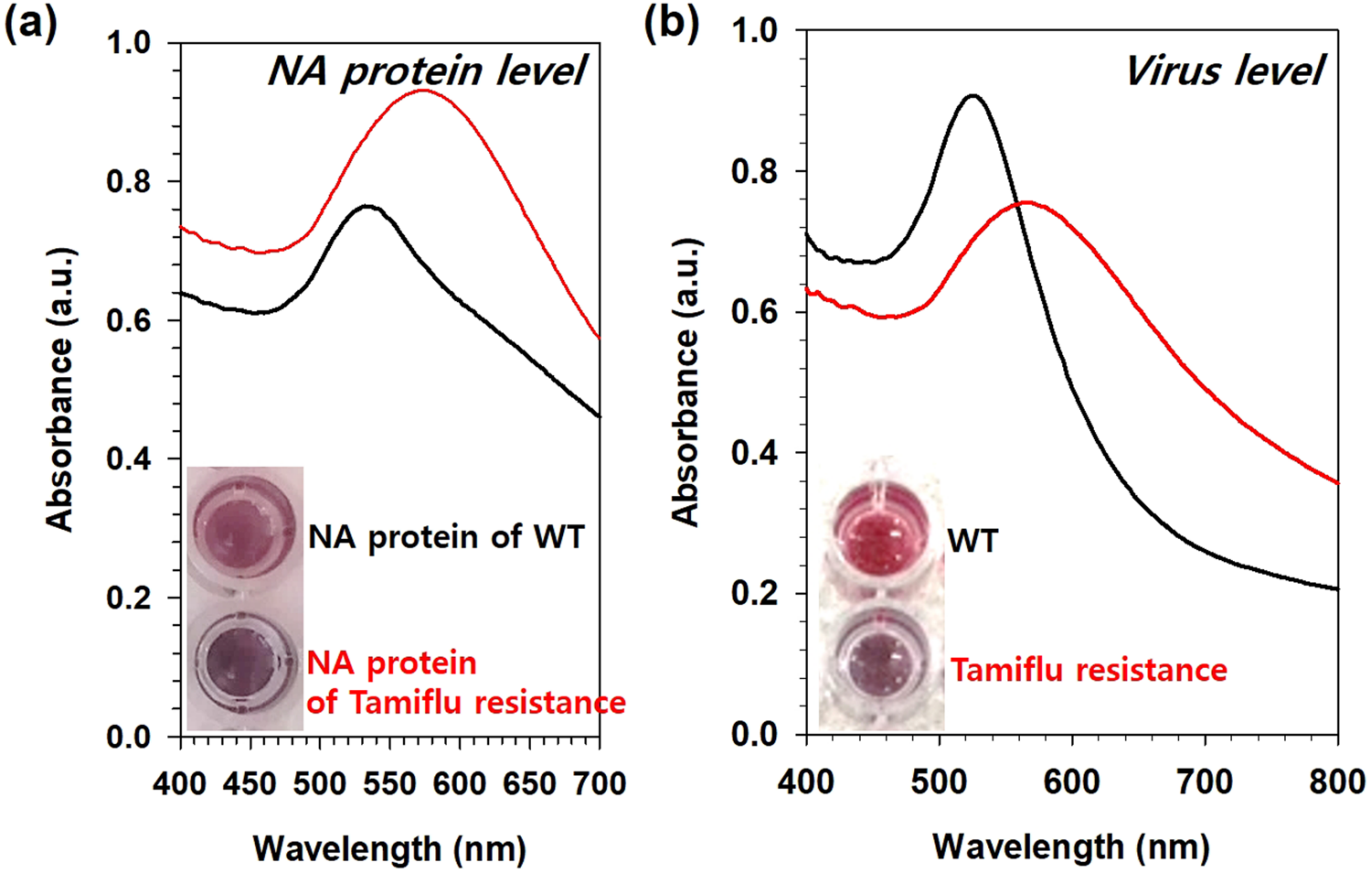
Colorimetric detection of OHT-GNPs against NA proteins and real viruses. OHT-GNPs were treated with (**a**) NA proteins (10 μg) and (**b**) real viruses (10^3^ PFU/test). After 5 min, their absorption profiles were measured using a multidetection microplate reader, and colorimetric changes were observed. The insert photos show the colorimetric changes (black: WT, and red: Tamiflu-resistant). The purple color indicates that OHT-GNPs detected Tamiflu resistance (or the NA protein of the Tamiflu-resistant virus) due to the binding with the Tamiflu-resistant enzyme.

**Figure 5:**
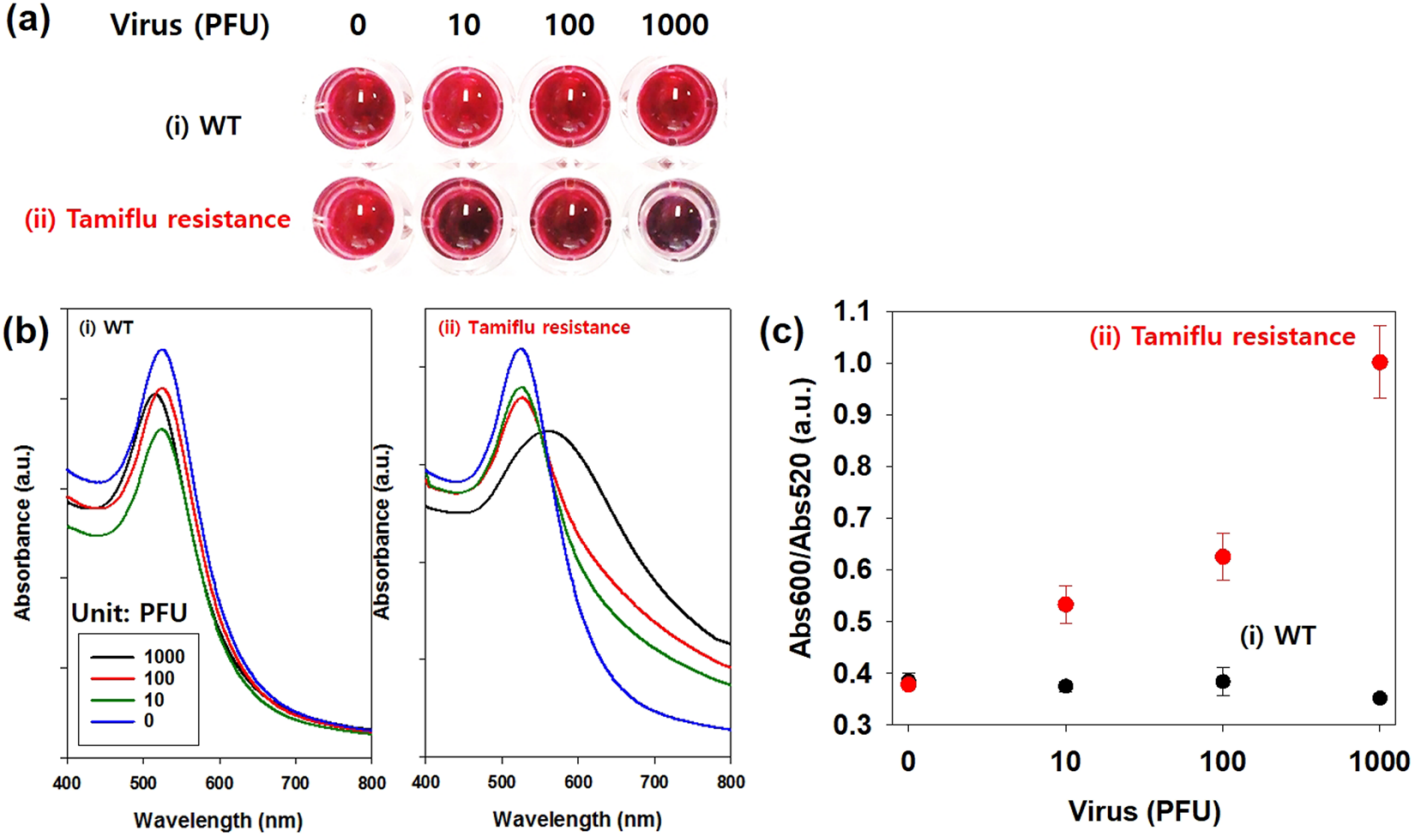
Sensitivity analysis of Tamiflu resistance detection using OHT-GNPs for colorimetric detection after a 5-min treatment of WT and Tamiflu-resistant viruses. (**a**) Images of the colorimetric changes and (**b**) absorption profiles of OHT-GNP solution in the presence of different virus concentrations (i: WT, and ii: Tamiflu-resistant). (**c**) The ratio of absorbances at 600 nm and 520 nm (Abs600/Abs520) (Abs: absorption) of (**b**). Circular dots and error bars indicate the averages and standard deviations from three independent experiments, respectively. This graph implies that the color of the OHT-GNP solution gradually changes to purple as the concentration of the Tamiflu-resistant virus increases, but the color of the OHT-GNP solution hardly changes when WT is present (PFU: plaque-forming units).

Next, using UV-Vis absorption spectroscopy, we examined the color change of the OHT-GNPs in the presence of the Tamiflu-resistant NA protein compared with that in the presence of the WT NA protein (Fig. 4(a)). Initially, the OHT-GNPs were deep red and exhibited a maximal absorption band at 520 nm. After the addition of the Tamiflu-resistant NA protein, the solution color rapidly (within 5 min) changed from red to purple, and the absorption spectrum was redshifted, exhibiting a maximal absorbance at approximately 600 nm, as shown in Fig. 4^24–30^. Thus, OHT-GNPs were able to interact with the NA protein structure, thereby decreasing the distance between the GNPs and causing the formation of OHT-GNP/NA complex. This aggregation was accompanied by a color change from red to purple. By contrast, neither an apparent color change nor an absorption spectral shift was observed when the WT NA protein was used as a control. Therefore, we further investigated the use of OHT-GNPs to detect the Tamiflu resistance with the naked eye (Fig. 4). When Tamiflu-resistant NA was added to the OHT-GNP solution, the solution color clearly changed to purple, indicating GNP aggregation because of their binding to the surface protein structure of the Tamiflu-resistant NA (Fig. 4(b)). In addition, their absorption band shifted from 520 nm to 600 nm and increased in intensity, reflecting GNP aggregation. In the presence of the WT NA, no color change or shift in the absorbance spectrum was detected (Fig. 4(a)). We also investigated the sensitivity of the OHT-GNP-based colorimetric assay for Tamiflu resistance at different virus concentrations. As shown in Fig. 5, the color of the OHT-GNP solution obviously changed from deep red to purple as the concentration of the Tamiflu-resistant virus increased because of OHT-GNP aggregation via the specific interaction between OHT and the NA structure (Tamiflu-resistant) on the virus. Simultaneously, the UV-Vis absorption spectrum also shifted, with the absorbance at approximately 520 nm (Abs520) decreasing and that at 600 nm (Abs600) increasing. By contrast, in the presence of the WT virus, OHT-GNP solutions largely did not change their color or characteristic absorption patterns (Fig. 5(a,b)). Based on these results, we plotted the ratio of absorbances at these two wavelengths (Abs600/Abs520) versus various virus concentrations for a quantitative analysis. Abs600 and Abs520 corresponded to purple-colored aggregates and red-colored dispersed particles, respectively. Thus, as the Abs600/Abs520 value increased, the solution became increasingly purple, indicating more aggregation. As shown in Fig. 5(c), whereas the Abs600/Abs520 ratio was constant regardless of WT concentration, this value exhibited a linear correlation with the concentration of the Tamiflu-resistant virus. Even at 10 PFU of the Tamiflu-resistant virus, the Abs600/Abs520 value was approximately 0.5. Therefore, using OHT-GNPs in solution, Tamiflu resistance could be directly detected with the naked eye based on color change or absorption spectral measurement at virus concentrations of 10 PFU or higher^24^.

### Tamiflu resistance detection using OHT-based LFA

**Figure 6:**
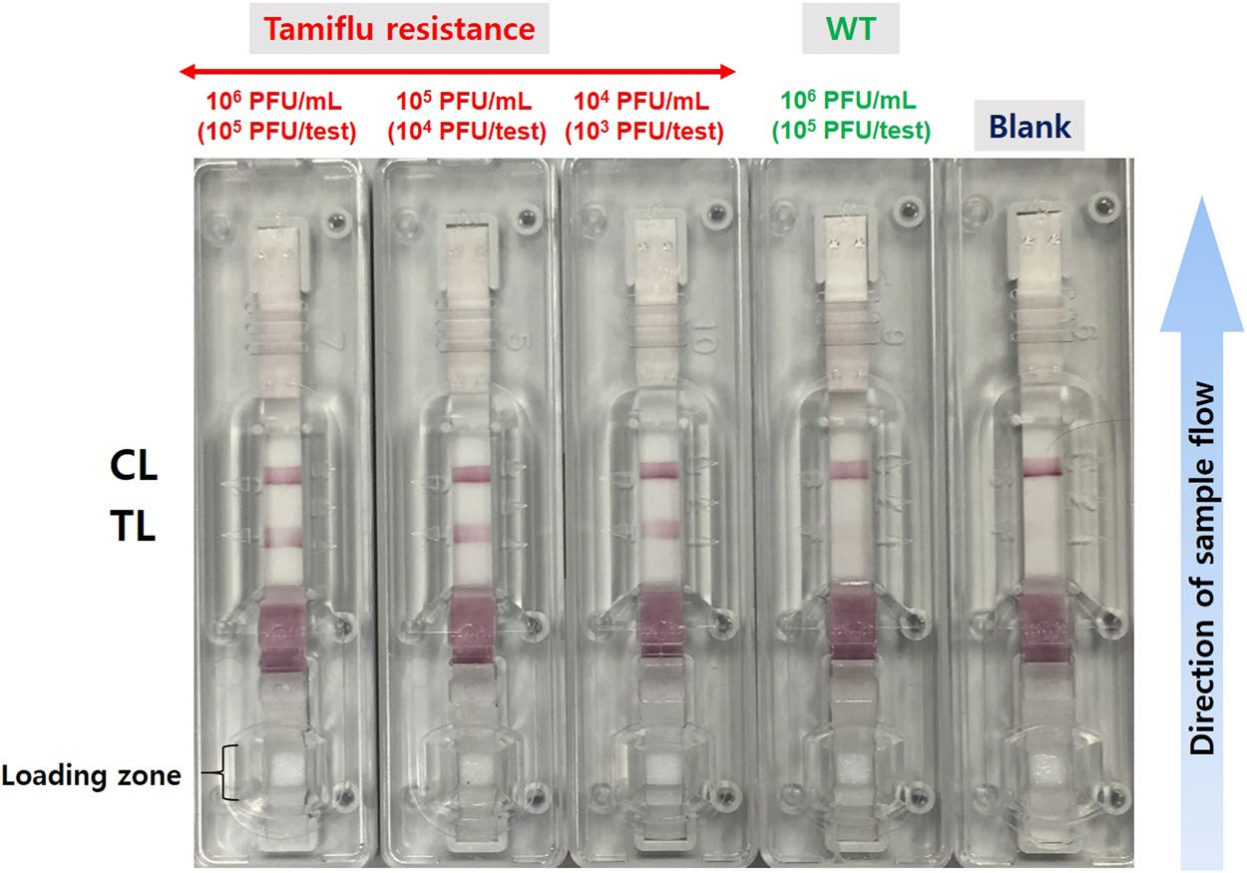
Sensitivity analysis of Tamiflu resistance detection using an OHT-based lateral flow assay (LFA) in response to various virus concentrations within 10 minutes. Blank and WT are used as controls. Virus-containing buffer (100 µL) is dropped onto a loading zone (sample pad) of the LFA, and then, this solution flows in the direction of the arrow. After 10 min, in the presence of Tamiflu resistance, two lines, TL and CL, appear. For the WT and blank, however, only one line appears at CL.

**Figure 7:**
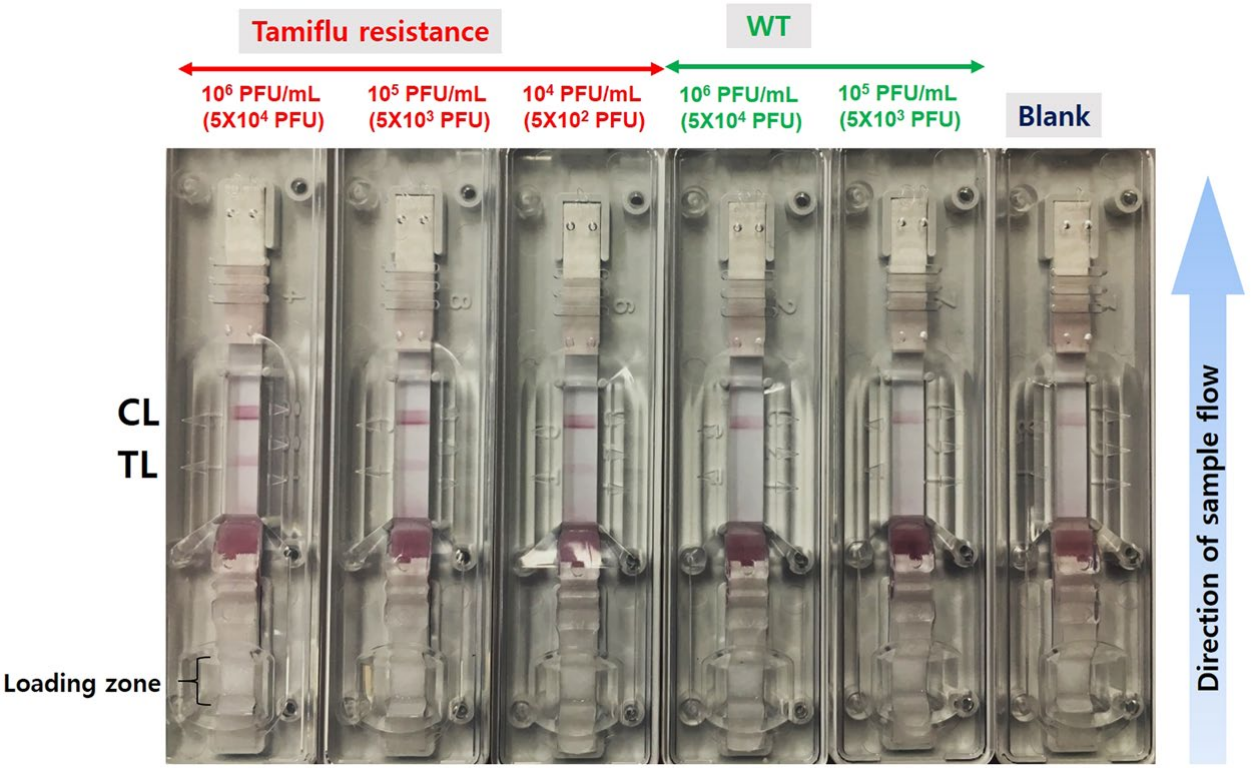
The sensitivity analysis of Tamiflu resistance detection using an OHT-based lateral flow assay (LFA) in response to various virus concentrations in 10% nasal fluid within 10 minutes. Blank and WT are used as controls. The virus (50 µL) is mixed with 10% nasal fluid-containing buffer, and then, this buffer is dropped into a loading zone (sample pad) of the LFA. After 10 min, in the presence of Tamiflu resistance, two lines at TL and CL appear. For the WT and Blank, however, only one line appears at CL.

**Figure 8:**
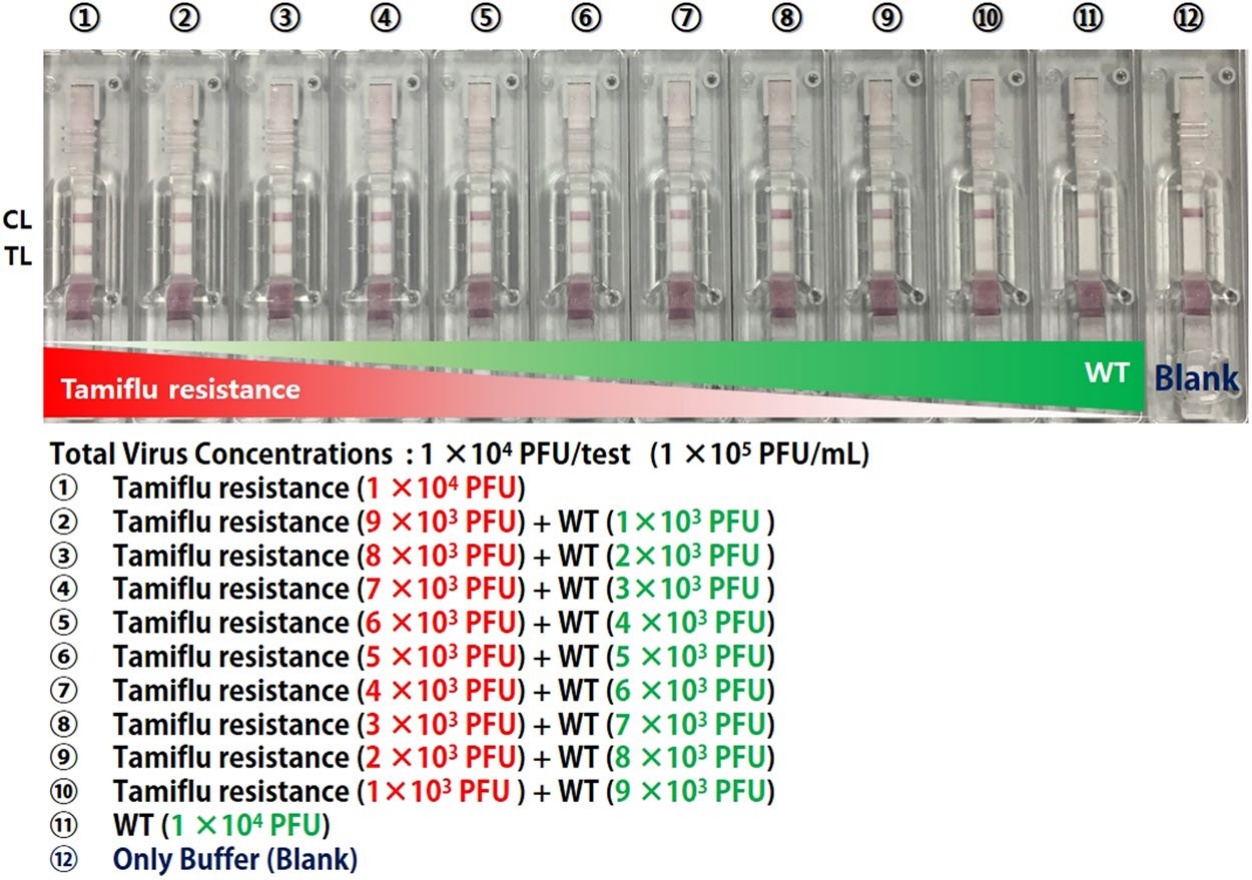
Specificity analysis of Tamiflu resistance detection using an OHT-based lateral flow assay (LFA) at various ratios of WT and Tamiflu-resistant viruses within 10 min. Mixed buffer containing Tamiflu-resistant and WT viruses is dropped onto a loading zone (sample pad) of the LFA. After 10 min, the test line (TL) appears darker as the concentration of the Tamiflu-resistant virus increases. Blank (buffer only) is used as a control. The control line (CL) indicates that this OHT-based LFA is operating correctly.

Additionally, a lateral flow assay as a POC device enables visual detection of the presence or absence of the Tamiflu-resistant virus using a rapid and simple diagnostic device (Fig. 1). OHT-GNPs bind to the Tamiflu-resistant virus because the NA site on the Tamiflu-resistant enzyme enables the complex formation; the OHT-GNP/H275Y mutant complex binds to antibody on the test line (TL), and the detection can be made based on the GNP color. By contrast, OHT of OHT-GNPs did not capture the WT virus; thus, the TL was not observed in the presence of the WT virus and in the absence of viruses (Fig. 6). Similarly, non-captured/excess OHT-GNPs continued to migrate toward the control line (CL), where they were captured by Tamiflu-resistant NA protein. Therefore, we observed both the TL and CL when the Tamiflu-resistant virus was detected, whereas only the control line was observed in the absence of the target virus. Using this OHT-based LFA, we further quantitatively detected the Tamiflu-resistant virus at various concentrations (10^6^ ~ 10^4^ PFU/mL). As shown in Fig. 6, TLs appeared in the presence of the Tamiflu-resistant virus and gradually dimmed as the concentration of the Tamiflu-resistant virus decreased. Notably, at 10^3^ PFU/test (=10^4^ PFU/mL) of Tamiflu-resistant virus, TL was clearly detected. However, with both the WT virus and blank conditions, only one line at the CL was observed, even at high concentrations (10^5^ PFU/test, 10^6^ PFU/mL) of WT virus. We also evaluated this OHT-based LFA under the 10% nasal fluid condition, to make it similar to actual clinical environments. TL appearance was caused by the nasal fluid containing Tamiflu-resistant virus. The TL in Fig. 7 appeared slightly blurrier than that in Fig. 6 because of nasal fluid; however, two lines were clearly visible at the TL and CL at 5 × 10^2^ PFU per test (=10^4^ PFU/mL) of the Tamiflu-resistant virus. For the WT virus, only one line was observed at CL, even at high concentrations (10^6^ PFU/mL). These results demonstrate that the OHT-based LFA can quickly and easily detect Tamiflu resistance. We further confirmed the detection specificity of the OHT-based LFA by generating mixtures with various ratios of WT and Tamiflu-resistant viruses. The total virus concentrations were 1 × 10^4^ PFU/test (=1 × 10^5^ PFU/mL), and only buffer was used as a control. As the concentrations of the Tamiflu-resistant virus decreased, the detection signals on the TL gradually declined from strong to weak (Fig. 8). The TL was observed even for a small amount of Tamiflu-resistant virus (1 × 10^3^ PFU) in the presence of a high amount of WT (9 × 10^3^ PFU) (Fig. 8 - ⑩). However, in the presence of only WT virus (1 × 10^4^ PFU) and buffer, the signal was observed only on the CL and not on the TL. These results have demonstrated that OHT-based LFA showed high detection specificity depending on the dose of the Tamiflu-resistant virus, with a 5 × 10^2^ ~ 10^3^ PFU/test (10^4^ PFU/mL) detection limit.

## Conclusions

We have developed a novel oseltamivir derivative (OHT) capable of specifically binding to NA protein in Tamiflu-resistant virus. Various OHT-based diagnostic assays could be developed to simply and rapidly detect Tamiflu resistance due to the specificity of OHT. OHT-GNPs can be used to detect Tamiflu-resistant NA over WT NA based on a color change. Moreover, OHT-based LFA as a rapid kit has confirmed the possibility of a qualitative as well as quantitative detection of viruses with a Tamiflu-resistant NA with the naked eye. This OHT-based platform should facilitate the development of rapid diagnostic platforms useful for POC detection of Tamiflu resistance.

## Electronic supplementary material

Supplementary information
