# Supplementary material for: Rapid and simple detection of Tamiflu-resistant influenza virus: Development of oseltamivir derivative-based lateral flow biosensor for point-of-care (POC) diagnostics
Source: Sci Rep. 2018 Aug 29;8:12999. doi: 10.1038/s41598-018-31311-x (PMC6115449; doi:10.1038/s41598-018-31311-x)
Supplement: Supplementary file 1 — Supplementary information [file 41598_2018_31311_MOESM1_ESM.doc]

Supplementary information

**Rapid and simple detection of Tamiflu-resistant influenza virus: Development of oseltamivir derivative-based lateral flow biosensor for point-of-care (POC) diagnostics**

Seul Gee Hwang^a,c,f^, Kab Ha^a,f^, Kyeonghye Guk^a,c^, Do Kyung Lee^b^, Gayoung Eom^d^, Sinae Song^a^, Taejoon Kang^a,c^, Hwangseo Park^e^, Juyeon Jung*^a,c^ and Eun-Kyung Lim*^a,c^

*^a^* Hazards Monitoring BioNano Research Center, Korea Research Institute of Bioscience and Biotechnology, 34141, Daejeon, Republic of Korea.

*^b^* BioNano Health Guard Research Center, Korea Research Institute of Bioscience and Biotechnology (KRIBB), 34141, Daejeon, Republic of Korea.

*^c^*Department of Nanobiotechnology, KRIBB School of Biotechnology, University of Science and Technology (UST), 125 Gwahak-ro, Yuseong-gu, Daejeon, 34113, Republic of Korea

^d^Department of Chemistry, KAIST, 291, Daehak-ro, Yuseong-gu, Daejeon, Korea

^e^Department of Bioscience and Biotechnology, Sejong University, Seoul 05006, Korea

*^f^*These authors contributed equally to this work.

*To whom correspondence should be addressed.

E-mail: eklim1112@kribb.re.kr (E.-K. Lim); jjung@kribb.re.kr (J. Jung)


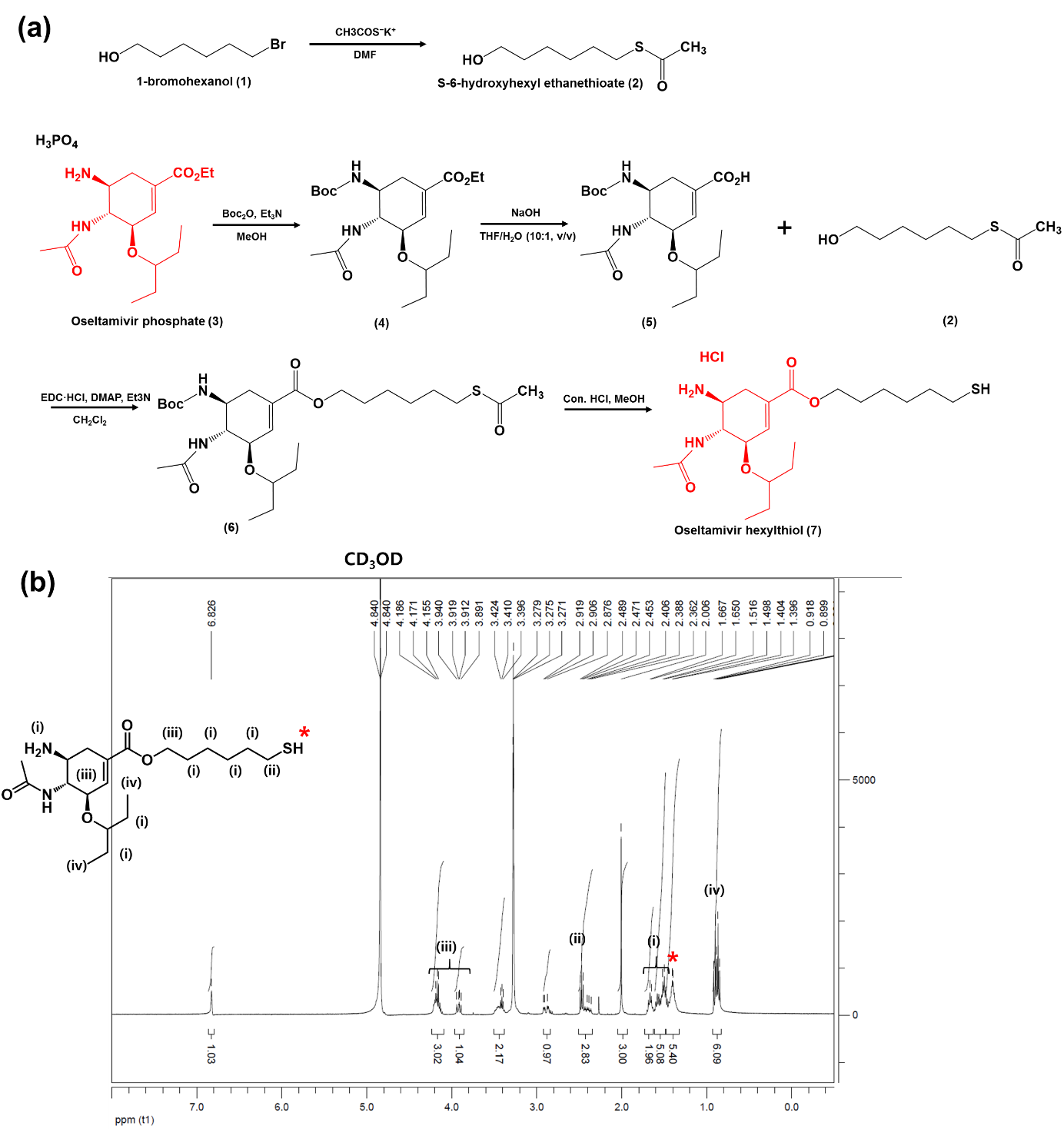


**Figure S1.** (a) Synthetic scheme, and (b) ^1^H-NMR spectra of oseltamivir hexylthiol (OHT) in CD_3_OD (*: thiol group (-SH)).


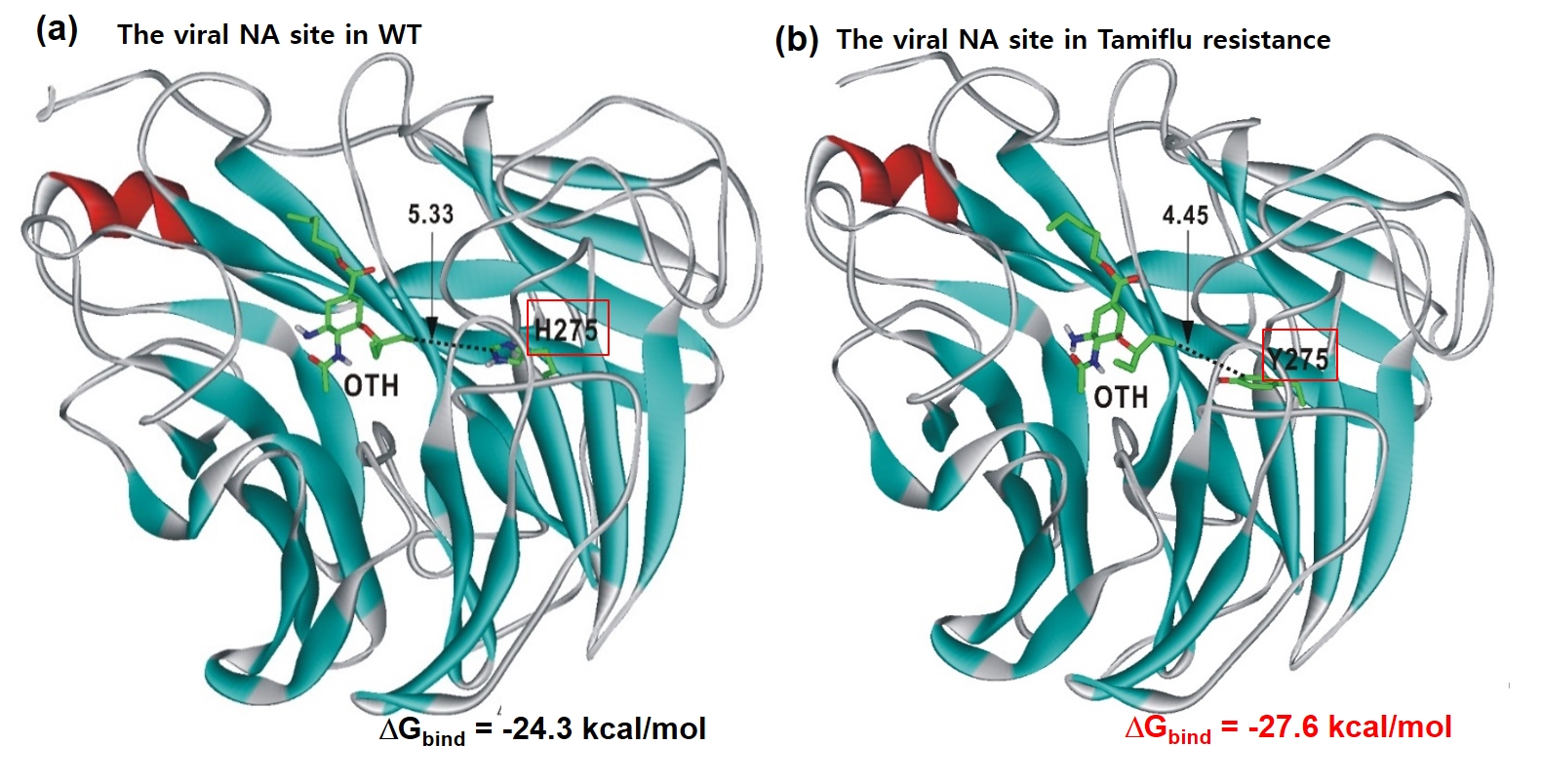


**Figure S2.** Comparative view of the docking poses of OHT in the NA site in (a) WT and (b) Tamiflu-resistance. Carbon atoms of OHT are indicated in green. The interatomic distances between the terminal 3-pentyl group of OHT and the aromatic rings of residue 275 are also shown.


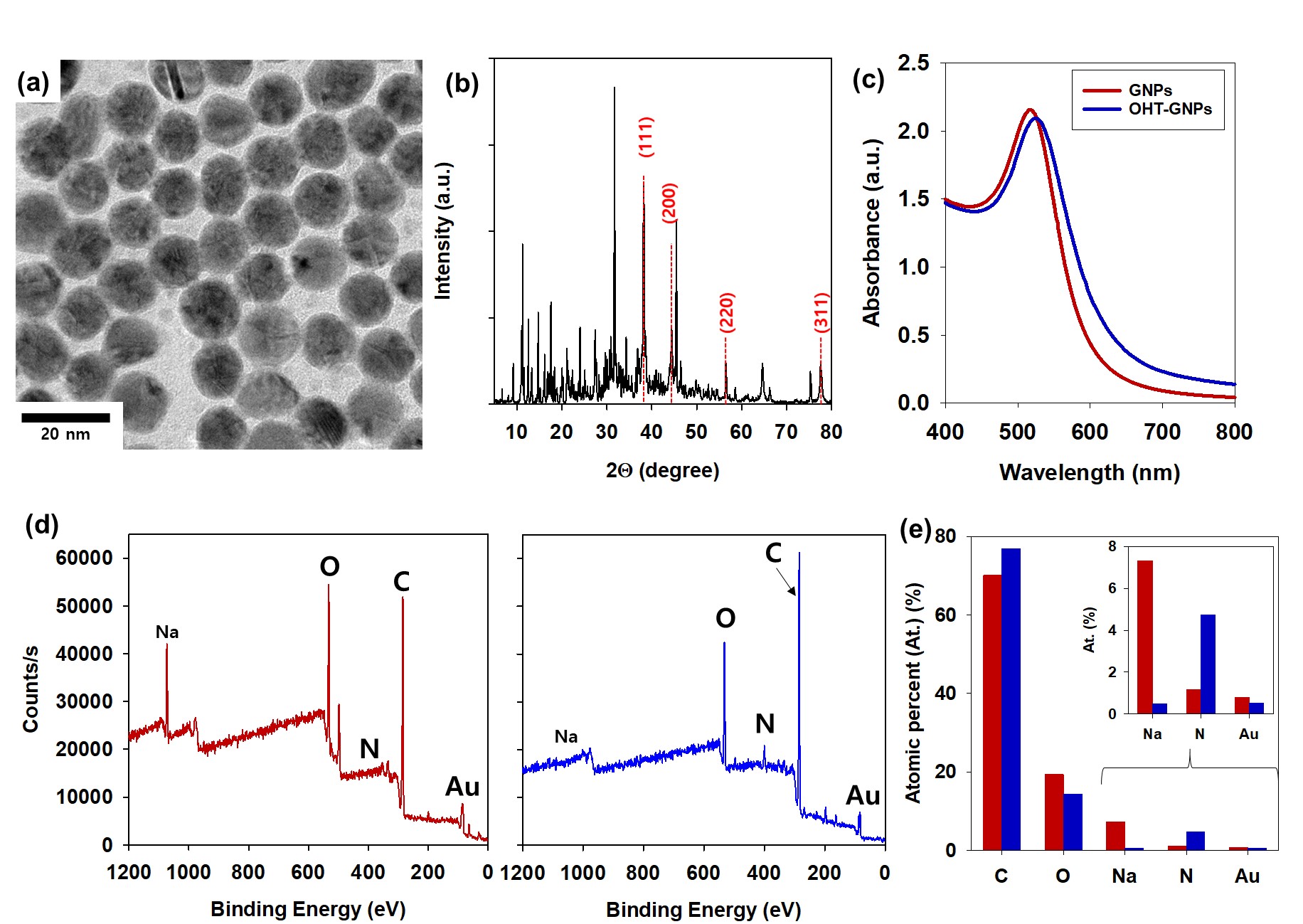


**Figure S3.** Characterization of gold nanoparticles (GNPs) and OHT-GNPs. (a) TEM image and X-ray diffraction (XRD) pattern of GNPs, (c) absorbance spectra of GNPs (deep red) and OHT-GNPs (deep blue), (d) the elemental composition on their surface and (e) their atomic percent (At.) by X-ray photoelectron spectroscopy (XPS) analysis (GNPs: deep red, and OHT-GNPs: deep blue).


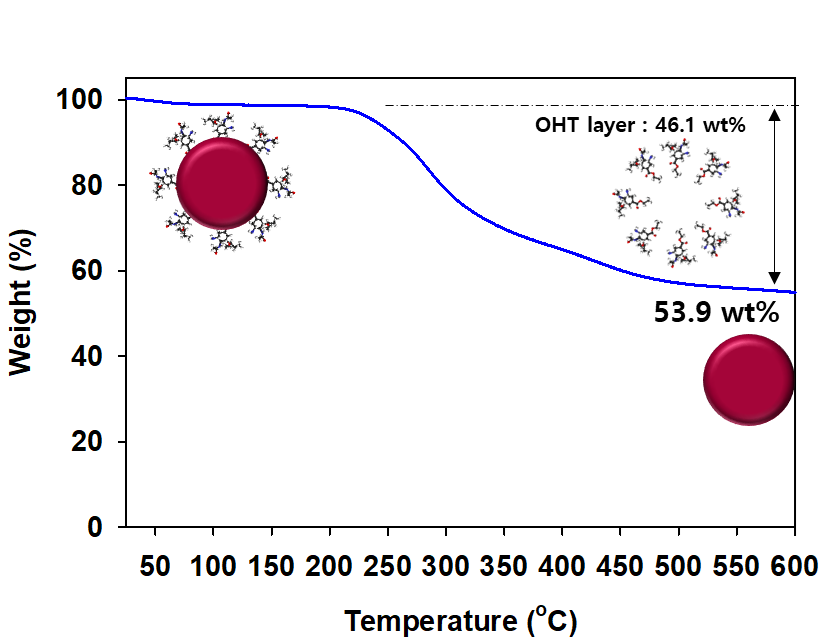


**Figure S4.** Thermogravimetric analysis (TGA) of OHT-GNPs. OHT-GNPs contains approximately 46.1% OHT and 53.9 % GNPs.
